# A Unique Case of Left Second Supernumerary and Left Third Bifid Intrathoracic Ribs with Block Vertebrae and Hypoplastic Left Lung

**DOI:** 10.1155/2013/620120

**Published:** 2013-12-12

**Authors:** Parag Suresh Mahajan, Islam Ali Hasan, Nazeer Ahamad, Nawal M. Al Moosawi

**Affiliations:** ^1^Department of Radiology, Hamad Medical Corporation, Doha, Qatar; ^2^Department of Radiodiagnosis, Armed Forces Hospital, Kuwait

## Abstract

Intrathoracic rib (IR) is a very rare anomaly in which a normal, an accessory, or a bifid rib lies within the chest cavity and may originate from a vertebra or a rib. It is more commonly present on the right side, and sometimes it may be associated with vertebral anomalies. Only 50 cases have been reported to date in the literature. In most cases, the IR is an isolated finding; it is incidentally detected and is asymptomatic. The IR can be easily missed on a chest radiograph and can be mistaken initially for a pleural lesion, lung consolidation, other peripheral lung parenchymal lesions, or a bony lesion. It is, therefore, essential for physicians and radiologists to know about this entity and consider it in the differential diagnosis, to avoid further evaluation and unnecessary investigations. We present a unique case of three intrathoracic ribs, a left second supernumerary rib, left third depressed normonumerary rib, and bifid arm of the left third rib, with block vertebrae and hypoplastic left lung. To our knowledge, this is the first such case presentation in the published literature.

## 1. Introduction

Ribs are protective ribbon-like bony elements normally present within the chest wall and are few of the most imaged structures in the clinical practice. The term “intrathoracic rib” signifies abnormal location of a rib within the thoracic cavity. The intrathoracic rib (IR) is a very rare congenital anomaly in which a normal rib, an accessory rib, or one arm of a bifid rib courses abnormally into the chest cavity [1]. It may originate from a vertebra or a rib, and sometimes it may be associated with vertebral anomalies [1]. It is more commonly present on the right side and between the third rib through the eighth rib, and no gender predilection is reported [2]. Only 50 cases have been reported till date in the literature [1–9].

## 2. Case Report

**Figure 1:**
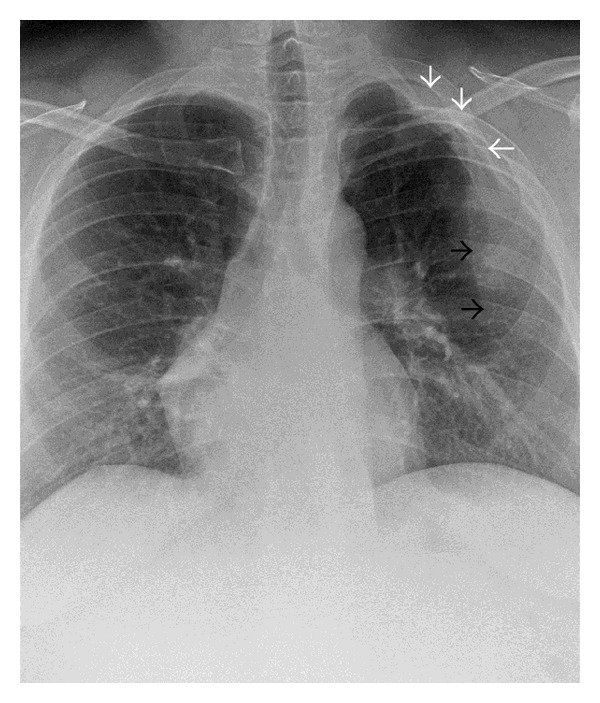
43-year-old gentleman complaining of high grade fever, cough, and chest pain was investigated with chest radiographs and helical chest CT scan and subsequently diagnosed with intrathoracic ribs. Chest radiograph shows left upper and mid-zone opacity (black arrows) and depression of bifid left third rib (white arrows). Upper lobe of the left lung is hypoplastic.

**Figure 2:**
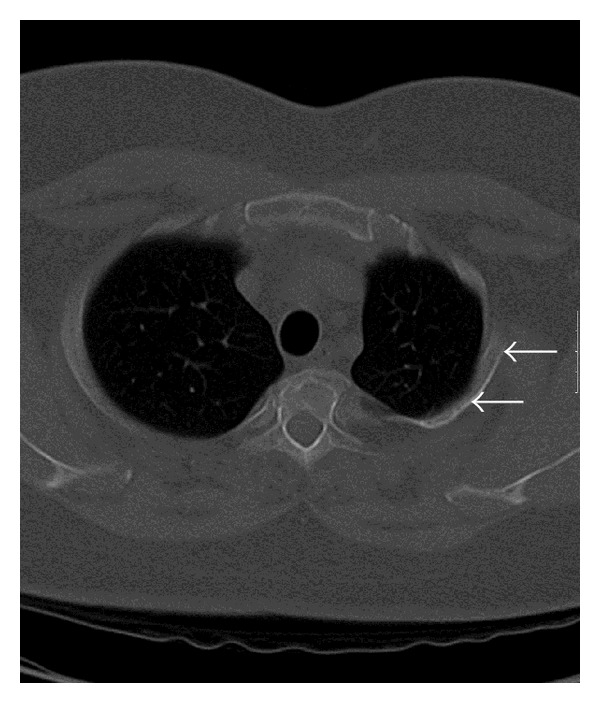
43-year-old gentleman complaining of high grade fever, cough, and chest pain was investigated with chest radiographs and helical chest CT scan and subsequently diagnosed with intrathoracic ribs. Unenhanced axial chest CT scan (bone window) shows depressed left third intrathoracic rib (arrows) and hypoplastic upper lobe of the left lung.

**Figure 3:**
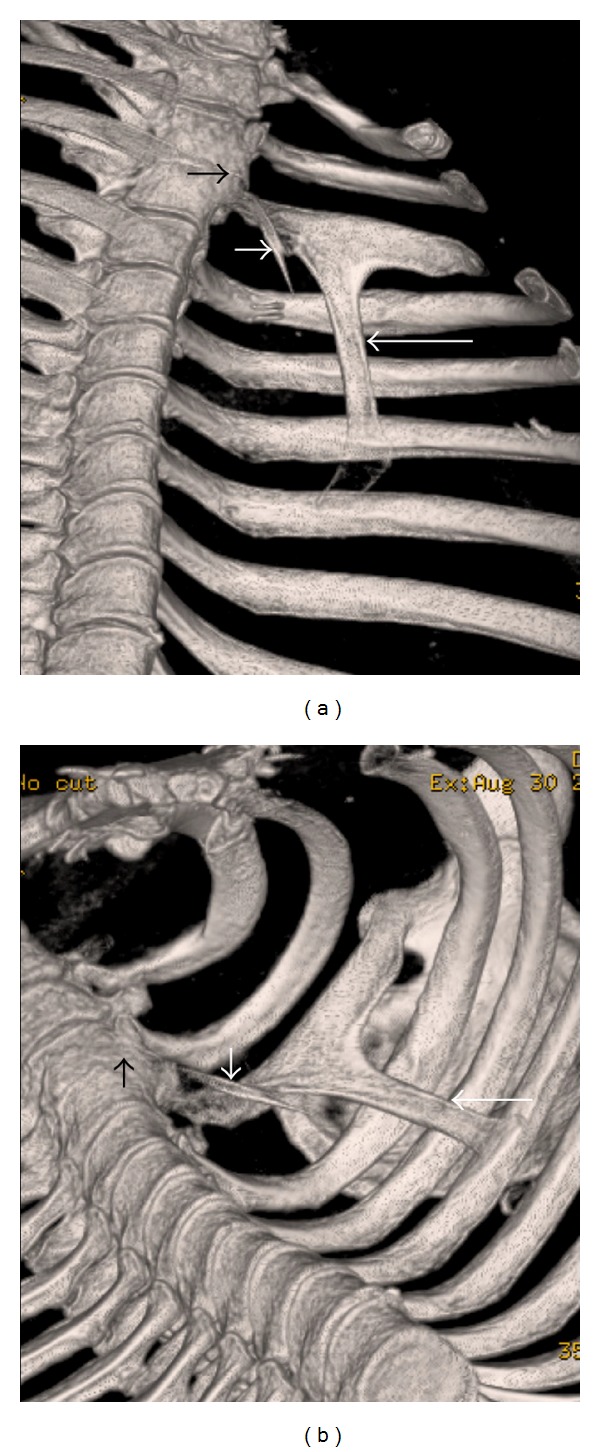
43-year-old gentleman complaining of high grade fever, cough, and chest pain was investigated with chest radiographs and helical chest CT scan and subsequently diagnosed with intrathoracic ribs. Unenhanced chest CT, volume rendered-3D images, coronal view in (a) and coronal oblique view in (b) reveal fusion of anterior aspects of the T2 and T3 vertebral bodies (short black arrow), a supernumerary second rib (thin, short, and obliquely oriented) with predominantly fibrous (non-ossified) proximal and distal parts originating from the second costovertebral neoarticulation (short white arrow), and the obliquely oriented bifid arm of the third rib coursing into the thoracic cavity (long white arrow).

**Figure 4:**
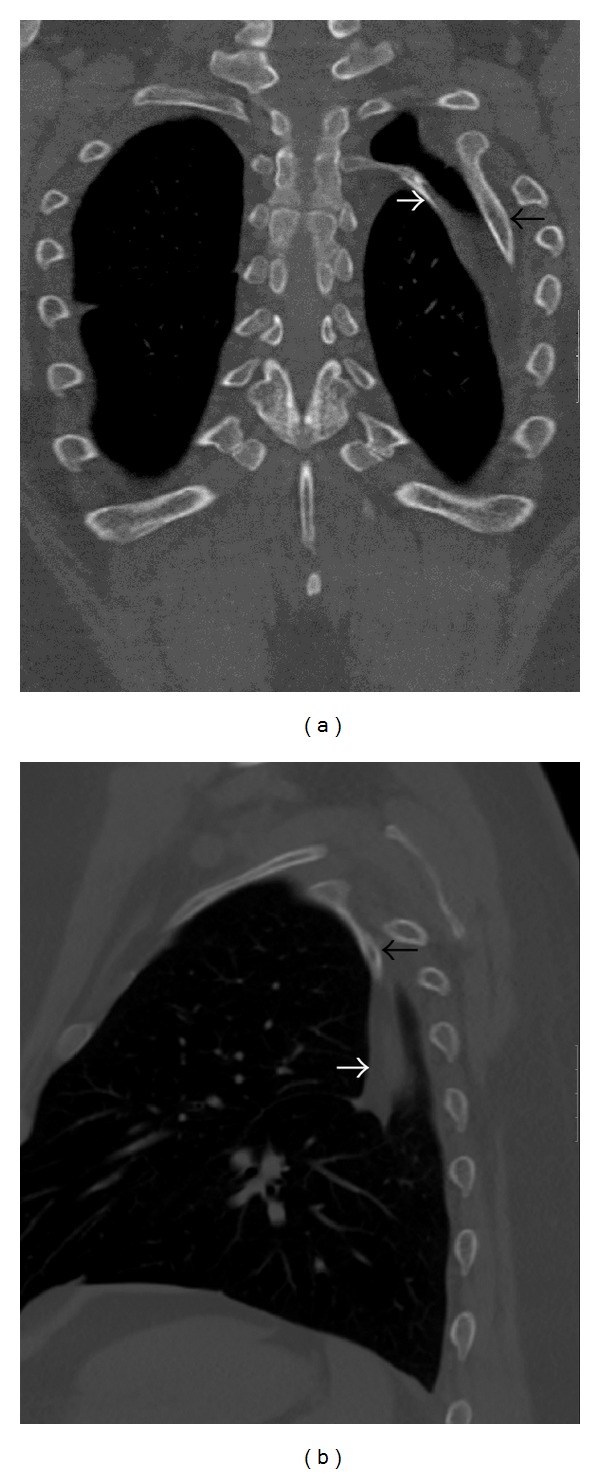
43-year-old gentleman complaining of high grade fever, cough, and chest pain was investigated with chest radiographs and helical chest CT scan and subsequently diagnosed with intrathoracic ribs. Unenhanced chest CT (coronal MIP reformatting, bone window) image in (a) shows left second supernumerary (white arrow) and left third bifid (black arrow) intrathoracic ribs, prominent extrapleural soft tissue around these ribs, and hypoplastic left lung. Unenhanced chest CT (sagittal MIP reformatting, bone window) image in (b) shows left third bifid intrathoracic rib (black arrow) and prominent extrapleural soft tissue around it (white arrow).

**Figure 5:**
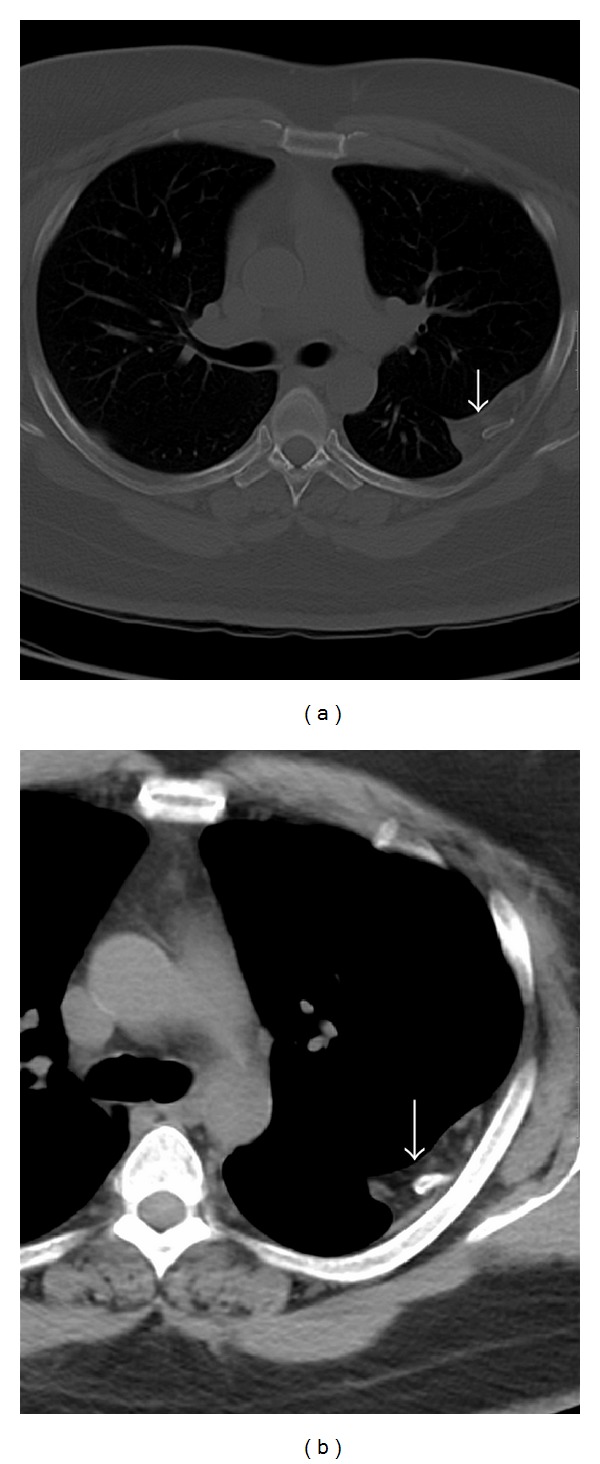
43-year-old gentleman complaining of high grade fever, cough, and chest pain was investigated with chest radiographs and helical chest CT scan and subsequently diagnosed with intrathoracic ribs. Unenhanced axial chest CT scans with bone window in (a) and mediastinal window in (b) show prominent extrapleural soft tissue including fat around the left intrathoracic rib (arrow).

A chest radiograph of a 43-year-old, Arab, gentleman was obtained as he complained of high grade fever, cough, and chest pain, of 12 hours duration. The chest radiograph revealed a triangular opacity in the left upper and mid zones laterally (Figure 1). Reduced air entry was noted on auscultation in the same region prompting possibility of lung consolidation, and the condition was medically managed. Two follow-up chest radiographs at 2-week intervals did not reveal any change in the left mid-zone opacity, although the patient was free of any symptoms. Additionally, depression of left third rib was noted leading to a suspicion of a rib abnormality (Figure 1). The patient had no other contributory medical history and specifically no history of chest trauma. Helical CT scan of the chest was performed for further evaluation and 3D, and multiplanar reconstructions were done. CT scan images demonstrate hypoplastic left upper lung lobe (Figure 2). CT scan images also reveal fusion of anterior aspects of the T2 and T3 vertebral bodies (Figures 3(a) and 3(b)). Two abnormal ribbon shaped bony structures are noted originating from the second and the third costovertebral articulations, respectively, on the left side (Figures 3(a) and 3(b)). The thin, short, and obliquely oriented structure originating from the second costovertebral articulation (separate from the second rib—neoarticulation) appears to be a supernumerary/accessory second rib coursing into the thoracic cavity. Its proximal and distal parts are predominantly fibrous (nonossified) (Figures 3(a) and 3(b)). The thick horizontally oriented depressed structure originating from the third costovertebral articulation appears to be a depressed normonumerary third rib (Figures 2, 3(a), 3(b), and 4(a)). It is shorter, thicker, and depressed into the chest cavity as compared to the contralateral normal third rib and is bifid as well. The obliquely oriented bifid arm of the third rib is thick and courses into the thoracic cavity (Figures 3(a), 3(b), and 4(b)). Prominent extrapleural soft tissue including fat is noted along the intrathoracic rib-like structures (Figures 5(a) and 5(b)). These features are suggestive of three intrathoracic ribs—a left second supernumerary rib, a left third depressed normonumerary rib, and the bifid arm of the left third rib associated with partial T3-T4 intervertebral fusion and hypoplastic left lung.

## 3. Discussion

Rib anomalies are relatively common and affect almost one percent of the general population [9]. Commoner rib anomalies include cervical ribs, bifid ribs, rib dysplasia, and intercostal fusion. IR is a very rare congenital anomaly; only 50 cases of IR have been reported till date in the literature after Lutz first described it in 1947 [1–9]. A third of these cases have been described in the pediatric population [2].

Although the embryological abnormality leading to the development of the IR is not conclusively known, it is suggested that it results from incomplete fusion of the cranial and the rostral segments of the sclerotome during embryological development. This likely occurs between fourth week and sixth week of fetal life. Abnormal gene expressions have also been implicated as a causative factor [1–3, 9].

Kamano et al. classified IRs into four types [9]. Type Ia is a supernumerary rib. Type Ib is a bifid rib originating from the posterior part of the rib. Type II is a depressed rib, and Type III is a bifid rib originating from the anterior part of the rib [9]. Of the 50 cases of intrathoracic ribs in the published literature, 26 can be classified as Type Ia, 15 as Type Ib, 2 as Type Ia+Ib, 1 as a Type I variant, 3 as Type II, 1 as Type III, and 2 as Type II+III. Our case has characteristics of Type Ia, Type II, and Type III (Type Ia+II+III) and thus to our knowledge, is the only such reported case to date [1–9].

Overall morphology of the intrathoracic rib resembles that of a normal rib. Prominent extrapleural soft tissue including fat is commonly detected around the intrathoracic rib to compensate for the deficiency caused by the chest wall deformity. Although a chest radiograph may help in the diagnosis, spiral CT scan is the modality of choice for diagnosing and evaluating the intrathoracic ribs [1–3].

In most cases, the intrathoracic rib is an isolated finding; it is incidentally detected and is asymptomatic [1, 2]. Although commonly asymptomatic, chest pain, breathing difficulty, and even blood in sputum have been reported in patients with intrathoracic ribs [1]. Symptoms are commoner in cases with adhesions between intrathoracic ribs and various organs [1, 4]. The intrathoracic rib can be easily missed on a chest radiograph and can be mistaken initially for a pleural lesion, lung consolidation, other peripheral lung parenchymal lesions, or a bony lesion. It is, therefore, essential for physicians and radiologists to know about this entity and consider it in the differential diagnosis, to avoid further evaluation and unnecessary investigations.

## 4. Conclusion

In conclusion, this is an extremely rare presentation of intrathoracic rib, as in our case three ribs, a supernumerary, an adjacent depressed normonumerary, and the bifid arm of the depressed rib, are intrathoracic; these are located on the left side and associated with corresponding vertebral anomalies and hypoplastic left lung. To our knowledge, this is the only such reported case to date.
